# Patterns of biopsy-proven renal diseases in geriatric patients: A single medical center experience

**DOI:** 10.1097/MD.0000000000031602

**Published:** 2022-11-18

**Authors:** Yung-Chieh Huang, Mei-Chin Wen, Ming-Ju Wu, Shang-Feng Tsai, Tung-Min Yu, Ya-Wen Chuang, Shih-Ting Huang, Shuo-Chun Weng, Mu-Chi Chung, Chia-Tien Hsu, Chun-Yi Wu, Chun-Te Huang, Tsai-Jung Wang, Hsien-Fu Chiu, Cheng-Hsu Chen

**Affiliations:** a Division of Nephrology, Department of Internal Medicine, Taichung Veterans General Hospital, Taichung, Taiwan; b Department of Pediatrics, Taichung Veterans General Hospital, Taichung, Taiwan; c Department of Pathology, Taichung Veterans General Hospital, Taichung, Taiwan; d School of Medicine, China Medical University, Taichung, Taiwan; e School of Medicine, Chung Shan Medical University, Taichung, Taiwan; f Institute of Clinical Medicine, School of Medicine, National Yang Ming Chiao Tung University, Taipei, Taiwan; g Center for Geriatrics and Gerontology, Taichung Veterans General Hospital, Taichung, Taiwan; h Ph.D. Program in Transplational Medicine, National Chung Hsing University, Taichung, Taiwan; i Department of Life Science, Tunghai University, Taichung, Taiwan; j College of Medicine, National Chung Hsing University, Taichung, Taiwan.

**Keywords:** end-stage kidney disease, geriatrics, glomerulonephritis, membranous nephropathy, nephropathy, renal biopsy

## Abstract

The elderly population is expanding rapidly, and that has become a major healthcare burden in terms of chronic kidney disease. The distribution patterns of kidney diseases in these elderly patients remain largely unclear. Here, we compared biopsy-based renal disease patterns between elderly and nonelderly patients. We performed a single-center, retrospective study (1992–2008) on biopsy-proven renal diseases to compare results between geriatric patients (age ≥ 65 years; n = 254) and nongeriatric patients (18 ≤ age < 65 years; n = 2592). Renal pathology was interpreted by pathologists based on light microscopy, immunofluorescence, and electron microscopy. The ages of the geriatric and nongeriatric groups were 71.8 ± 4.5 (65.1–87.3) and 39.7 ± 17.6 (18–64.9) years, respectively, and 74% and 41% of them, respectively, were men. In the geriatric group, the most frequent diagnosis was membranous nephropathy (46.1%), followed by minimal change disease/focal segmental glomerulosclerosis (16.9%), diabetic nephropathy (8.3%), hypertensive nephrosclerosis (7.5%), and IgA nephropathy (5.9%). The geriatric group had more membranous nephropathy and less lupus nephritis and IgA nephropathy than the nongeriatric group. Furthermore, the 5-year survival rate of the geriatric group was significantly low. Our results demonstrated the different distributions of renal biopsy patterns in geriatric patients diagnosed with acute or chronic progressive kidney injury and proteinuria through renal biopsy.

## 1. Introduction

According to the United States Renal Data System report, Taiwan is leading in terms of both incidence (493 per million population) and prevalence (3392 per million population) of treated end-stage kidney disease (ESKD).^[1]^ The commonest cause of treated ESKD is diabetes (45.4%). In 2017, 6969 (58.6%) of 11,887 patients who had dialysis were older than 65 years old.^[2]^ The National Development Council reported that the natural increase in the population is approaching zero, even with restricted international migration due to coronavirus disease 2019; the change in Taiwan’s population is expected to be negative beginning in 2020.^[3]^ In 2020, 16% of the people in Taiwan were > 65 years old. Now, the aging population is an important health care burden in Taiwan and even around the world. A serious concern is that Taiwan’s accelerated rate of aging even exceeds that of Western countries.^[4]^ In the elderly population, kidney health, including epidemiology and pathophysiology of chronic kidney disease, has been gaining research attention for treatment and management of the clinical manifestation of kidney disease.^[5,6]^ The risk of renal dysfunction is higher in geriatric patients than in young patients. The risk factors for renal dysfunction include conditions inherent with aging, such as gross structural and cellular changes, deterioration in physiological function, and lowered vascular compensatory reserve, and their worsening through exposure to nephrotoxic medications and diagnostic tests.^[6–8]^ Without renal biopsy, diagnosis and decision-making for elderly people are difficult in terms of the management of their primary and secondary glomerular diseases or other pathological entities.^[9,10]^

Indications for renal biopsy in elderly people are not formally defined. In theory, through biopsy, histopathological findings can be obtained regarding kidney injury severity, activity and chronicity of lesions, and the presence of other significant renal or vascular abnormalities. Despite this, physicians are uncertain about the risk–benefit ratio for elderly patients. Such uncertainty creates obstacles for physicians in deciding the order of renal biopsy.^[11]^ In an aging society, the performance of renal biopsies in geriatric patients to promote their kidney health is crucial to reduce kidney disease risk and to prolong life expectancy.^[11–13]^ The pattern of kidney disease distribution among geriatric patients is not well known in Taiwan. The aim of this study was to assess the etiology and clinical presentations of renal disease in elderly patients aged ≥ 65 years who underwent native renal biopsy based on our experiences in a medical center in central Taiwan.

## 2. Materials and methods

### 2.1. Study designs

This was a single-center retrospective study that included adult patients with biopsy-proven renal diseases at Taichung Veterans General from 1992 to 2008. The hospital is a medical center in central Taiwan. Patients who received renal biopsy after renal transplantation were excluded from this study. Among the enrolled patients, we identified 254 patients who were > 65 years old during their renal biopsy. They formed the geriatric group. Furthermore, we identified 2592 patients aged 18–64 years during their renal biopsy. They formed the nongeriatric group. Indications for biopsy were as follows: nephrotic syndrome, acute kidney injury (AKI), proteinuria without nephrotic syndrome, prolonged azotemia, nephritic syndrome, and chronic kidney disease with active urine sediment. For each group, we analyzed renal biopsy results and clinical information, including patient’s age, sex, initial serum creatinine (sCr), serum albumin and total protein, IgG, IgM, IgA, C3, C4, 24-h daily urine protein (DUP), creatinine clearance rate (CCr), urine protein/creatinine ratio, serum and urine protein electrophoresis, total blood count, and renal ultrasound data. Our study protocol was approved by the hospital’s ethics committee (IRB No. CE15125B). Due to the retrospective nature of the study data, the requirement of patients’ informed consent was waived.

### 2.2. Pathologic diagnosis

All renal biopsy specimens were obtained by hospital nephrologists by using biopsy needles under the guidance of sonography or computed tomography. Pathologists made the final diagnosis after reviewing renal tissue images obtained using the imaging modalities of light microscopy, immunofluorescence, and electron microscopy.

### 2.3. Statistical analyses

All analyzed data were stored in a standard EXCEL file. Statistical analysis was performed using SPSS version 13.0 for Windows (SPSS Inc., Chicago, IL). During the evaluation of study variables, descriptive indices (mean, standard error, and rate) were used. Comparisons were made using Student’s *t* test, Mann–Whitney *U* test, chi-square test, and Fisher’s exact test, as appropriate. Statistical significance was set at *P* < .05.

## 3. Results

### 3.1. Clinical characteristics

The clinical characteristics of included patients are shown in Table 1. The mean ages of patients during their renal biopsy were 71.8 ± 4.5 (65.1–87.3) and 39.7 ± 17.6 (18–64.9) years for the geriatric and nongeriatric groups, respectively. Furthermore, the proportions of men in the geriatric and nongeriatric groups were 74% and 41%, respectively, and the intergroup difference was statistically significant (*P* < .001). Renal functions of patients were worse in the geriatric group than in the nongeriatric group in terms of their initial sCr (3.1 ± 3.3 vs. 2.5 ± 4.4 mg/dL; *P* = .014), initial CCr (40.7 ± 29.5 vs. 67.1 ± 41.2 mL/min; *P* < .001), and DUP (5.4 ± 4.7 vs. 4.1 ± 4.4 g/day; *P* < .001). Furthermore, serum IgG, IgA, and C4 levels were significantly higher in the geriatric group than in the nongeriatric group (Table 1).

**Table 1:** The clinical characteristics of included patients.

	Geriatric (n = 254)	Nongeriatric (n = 2592)	*P* value
Age (yr)	71.8 ± 4.5	39.7 ± 17.6	<.001
Male gender n (%)	242 (74%)	978 (41%)	<.001
Initial Cr	3.1 ± 3.3	2.5 ± 4.4	.014
CCr (mL/min)	40.7 ± 29.5	67.1 ± 41.2	<.001
DUP (g/d)	5.4 ± 4.7	4.1 ± 4.4	<.001
IgG (mg/dL)	1178.9 ± 989.6	1007.7 ± 616.4	.026
IgA (mg/dL)	298.5 ± 179.5	270.5 ± 179.5	.028
IgM (mg/dL)	116.0 ± 105.2	116.5 ± 80.4	.471
C3 (mg/dL)	102.0 ± 29.9	91.2 ± 122.9	.114
C4 (mg/dL)	25.3 ± 11.3	18.7 ± 14.1	<.001

CCr = creatinine clearance rate, DUP = daily urine output.

### 3.2. Pathology findings

The most common diagnosis in the geriatric group was membranous nephropathy (MN; 46.1%) (Table 2), followed by minimal change disease/focal segmental glomerulosclerosis (MCD/FSGS) (16.9%), diabetic nephropathy (8.3%), hypertensive nephrosclerosis (7.5%), and IgA nephropathy (IgAN) (5.9%). Five patients with MN were cancer related. Furthermore, the most common diagnosis in the nongeriatric group was lupus nephritis (LN; 41.6%), followed by IgAN (17.9%), MCD/FSGS (17.1%), MN (14.5%), and diabetic nephropathy (3.4%).

**Table 2:** The distributions of pathological diagnoses of the geriatric and nongeriatric groups.

	Geriatric n (%)	Nongeriatric n (%)
MN	117 (46.1)	375 (14.5)
IgAN/MesPGN	15 (5.9)	465 (17.9)
MCD/FSGS	43 (16.9)	442 (17.1)
Crescent GN	10 (3.9)	37 (1.4)
lupus nephropathy	14 (5.5)	1077 (41.6)
Diabetic nephropathy	21 (8.3)	89 (3.4)
myeloma/amyloidosis	15 (5.9)	29 (1.1)
Ischemic change	19 (7.5)	78 (3.0)

The distribution was significantly different between the geriatric and nongeriatric groups (*P* < .001). MN, membranous nephropathy; IgAN, IgA nephropathy; MesPGN, mesangial proliferative glomerulonephritis; MCD, minimal change disease; FSGS, focal segmental glomerulosclerosis; GN, glomerulonephritis.

**Figure 1.**
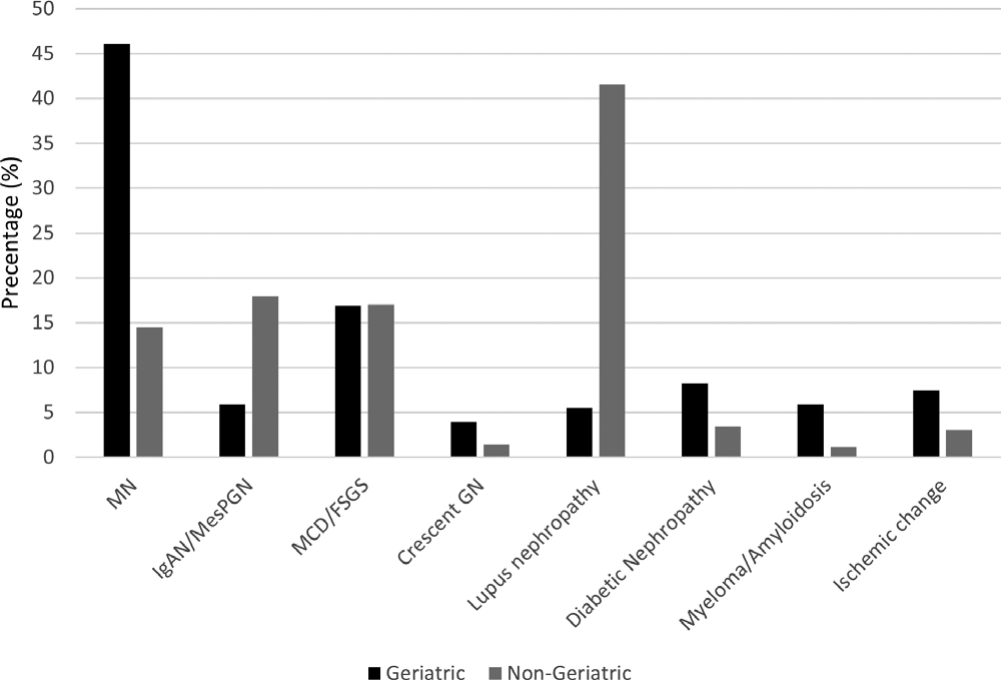
Pathology diagnosis distribution in the geriatric and nongeriatric groups.

The distributions of pathological diagnoses were significantly different between the geriatric and nongeriatric groups, with a *P* value of < 0.001 (Fig. 1). Compared with the nongeriatric group, more people in the geriatric group had MN (*P* < .01). However, LN (*P* < .001) and IgAN (*P* < .001) were less frequent in the geriatric group than in the nongeriatric group.

### 3.3. Survival

**Figure 2.**
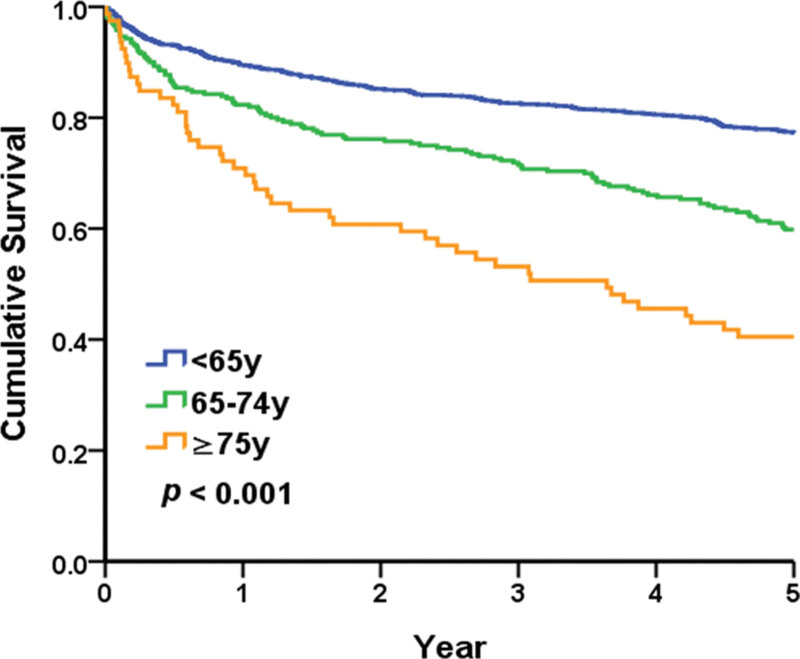
Survival curves of the geriatric and nongeriatric groups.

The 5-year survival rates in the geriatric and nongeriatric groups were 58.2% and 77.3%, respectively. In the geriatric group, the 5-year survival rates in those aged 65–74 and > 75 years were 59.8% and 40.5%, respectively. The survival curves were significantly different (Fig. 2).

**Figure 3.**
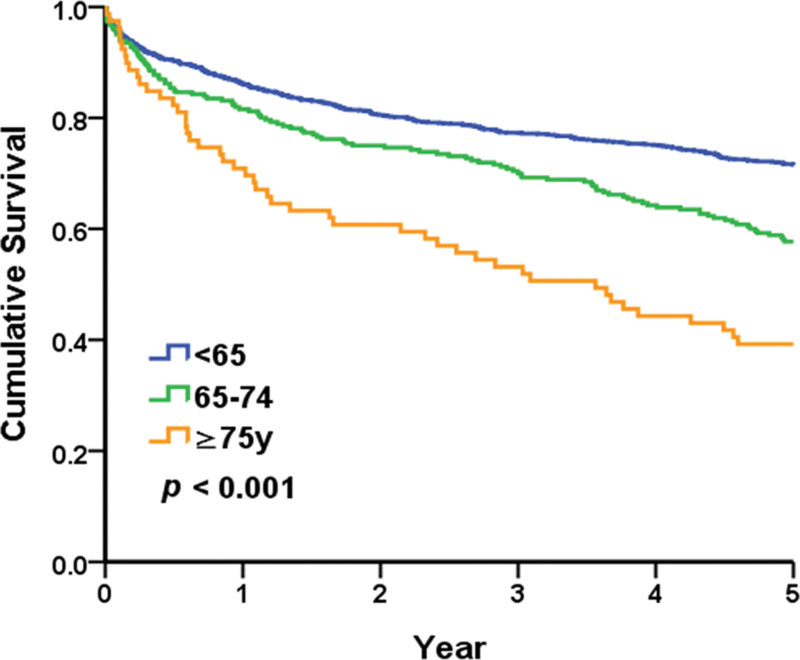
Renal survival curves of the geriatric and nongeriatric groups.

**Figure 4.**
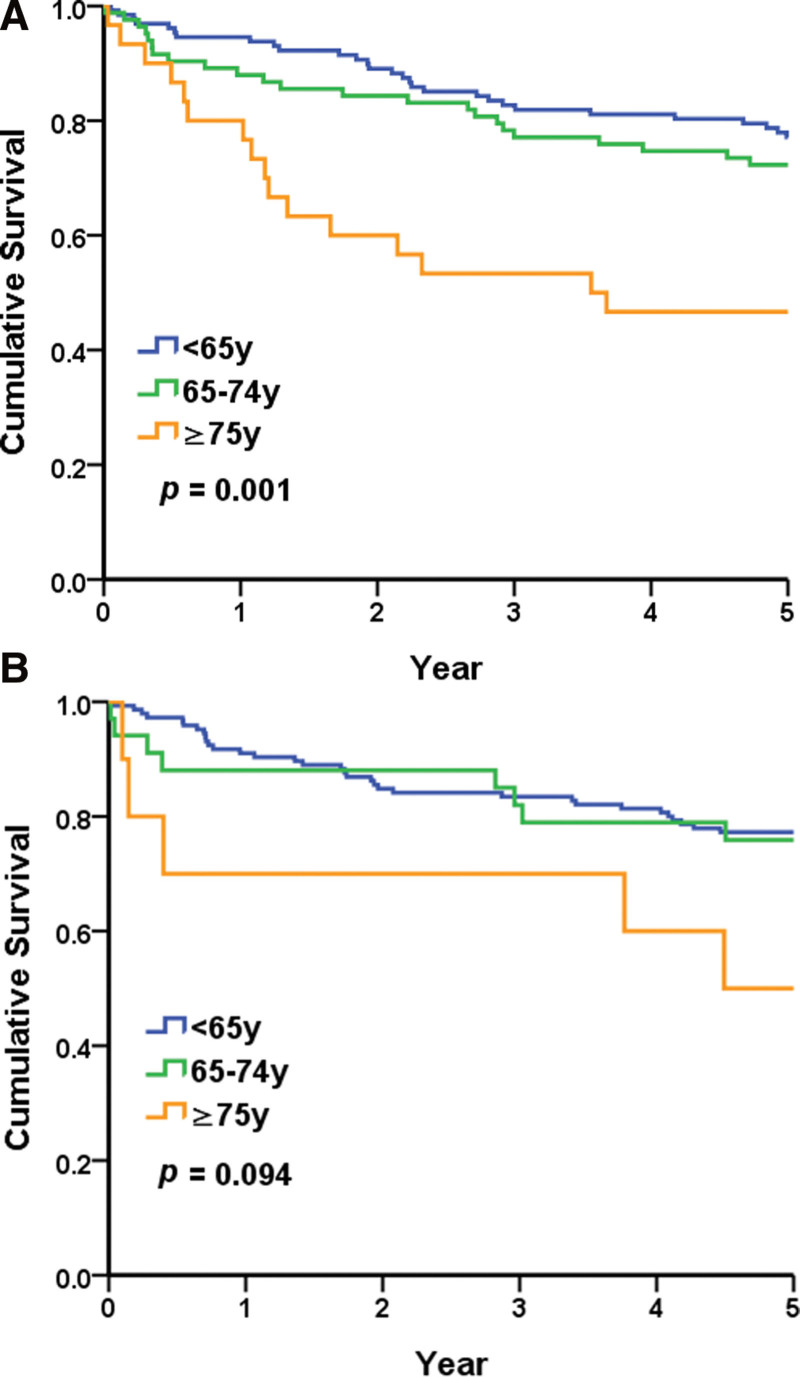
Renal survival curves for (a) membranous nephropathy and (b) minimal change disease/focal segmental glomerulosclerosis in the geriatric and nongeriatric groups.

The 5-year renal survival rates (including death and dialysis) in the geriatric and nongeriatric groups were 53.5% and 71.5%, respectively. In the geriatric group, the 5-year survival rates in those aged 65–74 and > 75 years were 57.7% and 39.2%, respectively. The survival curves were significantly different (Fig. 3). In patients with MN, 5-year renal survival rates in the geriatric and nongeriatric groups were 65.5% and 77.1%, respectively (*P* = .035). In patients with MCD/FSGS, 5-year renal survival rates in the geriatric and nongeriatric groups were 70.5% and 77.5%, respectively (*P* = .334). The survival curves of MN and MCD/FSGS subgroups are provided in Figs. 4A and B.

## 4. Discussion

In our study, pathological diagnosis distributions differed greatly between the geriatric and nongeriatric groups. LN was an important factor. Approximately 40% of the patients with systemic lupus erythematosus (SLE) had LN. Most patients with LN had received renal biopsy unless contraindicated because renal biopsies are essential for treatment plans and prognosis of patients with SLE. SLE is common among women of childbearing age. This accounted for the great differences in histological findings between the 2 groups. SLE patients are likely to have low serum C3 and C4 levels, particularly in those with high disease activity. Thus, the nongeriatric group could have reduced C3/C4 levels.

MN was the most common diagnosis in our geriatric group, and it is also the most prevalent diagnosis in the elderly according to the literature^[7,14]^; it accounts for ~32% of all biopsies. However, some recent studies have reported different findings. Perkowska-Ptasinska et al reported that MCD/FSGS was more common than MN (23.6% vs. 18.2%)^[15]^; Harmankaya et al reported that amyloidosis was the most common (18.4%) among all histological diagnoses.^[12]^ Recent studies have suggested that AKI is a common indication for biopsy,^[8]^ which might partially explain the discrepancy in the reported distributions of histological findings.

In this study, we also found that serum IgG and IgA levels were higher in the geriatric group than in the nongeriatric group. In the literature, serum IgG, IgA, and C3/C4 levels were never recorded for renal biopsy in elderly patients, and neither were renal biopsies of elderly and nonelderly populations compared.

The initial renal functions, measured as either sCr or CCr, were worse in the geriatric group than in the nongeriatric group. Considering the changes in renal function in the aging process even in healthy people, our findings are reasonable. The mean annual estimated glomerular filtration rate loss as estimated from the creatinine level was 0.4 ± 3.6 mL/min/1.73 m.^[16]^ Similar findings were reported by other studies focusing on the comparison of geriatric and nongeriatric populations.^[16,17]^

Nevertheless, no previous study directly compared 24-h DUP between geriatric and nongeriatric patients. The prevalence of proteinuria varied between different studies. In a multicentered study conducted in Poland, 55.6% of the geriatric patients had nephrotic proteinuria, which was significantly more than that in the younger group.^[15]^ In a single-center study in Japan, 60.2% of all geriatric patients had nephrotic range proteinuria.^[18]^

In our study, the 5-year survival rate was significantly lower in the geriatric group than in the nongeriatric group. No previous study on renal biopsy compared long-term survival between the elderly and general populations. Age itself is definitely one of the most important factors affecting survival time^[19]^; other factors, such as major comorbidities, self-care abilities, and the pathological diagnoses of renal biopsies, may contribute to differences in survival between the 2 groups.

In our study, the 5-year renal survival rate in the geriatric group was 53.5%. Aging involves macro- and micro-structural changes in the kidney. Aging-related changes in kidneys grossly include reduced cortical volume and the presence of cysts. The microanatomical changes in the kidney with old age involve a decreased number of functional glomeruli with more nephrosclerosis, including arteriosclerosis, glomerulosclerosis, and tubular atrophy, with interstitial fibrosis. The glomerular filtration rate declines at a rate of 6.3 mL/min/1.73 m^2^ with every 10 years of age.^[20]^ This may explain the reduced renal survival rate in the geriatric group generally. Nevertheless, our study found no significant differences in renal survival between the geriatric and nongeriatric groups with specific pathological diagnoses of MCD/FSGS.

MN accounted for nearly half of the pathological findings in our geriatric patients. A few studies have investigated long-term renal outcomes in patients with MN. Shiiki and colleagues^[21]^ focused on idiopathic MN with nephrotic syndrome. Their multivariate analysis suggested that the renal survival rate was lower in patients > 60 years old than in patients < 40 years old. In a retrospective survey, Choi and colleagues^[22]^ compared older and younger patients with idiopathic MN. In a multivariate analysis, they found that older age at biopsy is a poor predictor of patient renal survival, although the effect was nonsignificant (hazard ratio: 1.04, 95% confidence interval: 1.01–1.07). However, patients included in the study had better renal function (sCr 1.0 ± 0.8 and 1.2 ± 0.9 mg/dL in patients < 65 and ≥ 65 years old, respectively). However, Cattran and colleagues^[23]^ observed that older age (>50 years old) was associated with a significantly reduced ESKD risk. Conversely, patients with idiopathic MN had a high risk of cardiovascular events similar to those with ESKD, with a 5-year cumulative incidence of up to 8.8% in the study reported by Lee and colleagues.^[24]^ Due to that this was a retrospective study of an earlier period (1992–2008), most patients did not have data for phospholipase A2 receptor antibodies, which were discovered in 2009.^[25]^

Several studies have discussed the pros and cons of renal biopsy in the elderly population.^[9,10]^ Major and minor complications and diagnostic adequacy did not differ significantly between elderly and younger patients in 2 observational studies,^[10,12]^ but solid evidence, like studies using a case–control double-blind design or a large patient sample, is difficult to obtain. Almost all current studies on renal biopsy of elderly patients are based on retrospectively collected data. Many elderly people with acute or chronic kidney disease might never be considered for renal biopsy.

Our study has some limitations. First, decisions to perform renal biopsy were based on clinicians’ judgments and the willingness of patients and their families rather than on a standard protocol. Geriatric patients delay their renal biopsy until they reach advanced stages of glomerular sclerosis and tubular atrophy or develop interstitial fibrosis, which increases the difficulty and efficacy of management, considering the age, fragility, and immune risk. Second, many geriatric patients with AKI or nephrotic syndrome might have received their diagnoses through renal biopsy, which might have altered their therapy; however, the number of such patients in this cohort may not be large enough to demonstrate the long-term benefit of management in individual renal glomerulonephritis. This is a single center-based study with a relatively small number of biopsies, and our results may only represent as regional experience. Although the biopsy findings could be beneficial for patients with kidney diseases through a potential change in treatment, geriatric patients with fragile kidneys are vulnerable to hemodynamic changes and underdiagnosis of their disease entities, missing their golden therapeutic periods.

## 5. Conclusions

In this study, we found that histological findings were significantly different between the geriatric and nongeriatric groups. In our study, nearly half of the pathological findings in the geriatric group were MN, where LN was the most common diagnosis in the younger group. General survival and renal survival rates were lower in the geriatric group than in the nongeriatric group. In subgroups with different pathological diagnoses, a significant difference was observed between the 2 groups among those with MN, but not among those with MCD/FSGS.

Our results demonstrated the different distributions of glomerulonephritis patterns in geriatric patients diagnosed with acute or chronic progressive kidney injury and proteinuria through renal biopsy, and the identification of early treatment strategies for patients at risk of developing renal insufficiency might improve their outcome in the future. We proposed that for elderly patients, renal biopsy, as a valuable diagnostic tool, should be offered given its potential to beneficially modify treatment. Age should not be considered a contraindication to renal biopsy.

## Acknowledgments

The funder had no role in any part of this study. This manuscript was edited by Wallace Academic Editing.

## Author contributions

Conceptualization: YC Huang, CH Chen and MJ Wu; Methodology: YC Huang and CH Chen; Formal analysis: SF Tsai, TM Yu, YW Chuang, ST Huang, SC Weng, MC Chung, CT Hsu, CY Wu, CT Huang, TJ Wang, HF Chiu; Investigation of pathology: MC Wen; Data curation: MC Wen; Writing—original draft preparation: YC Huang and CH Chen; Writing—review and editing: SF Tsai, TM Yu, YW Chuang, ST Huang, SC Weng, MC Chung, CT Hsu, CY Wu, CT Huang, TJ Wang, HF Chiu; Supervision: CH Chen and MJ Wu; Funding acquisition: YC Huang and CH Chen.

**Conceptualization:** Yung-Chieh Huang, Ming-Ju Wu, Cheng-Hsu Chen.

**Funding acquisition:** Yung-Chieh Huang, Cheng-Hsu Chen.

**Methodology:** Yung-Chieh Huang, Cheng-Hsu Chen.

**Writing—original draft:** Yung-Chieh Huang, Cheng-Hsu Chen.

**Writing—review and editing:** Yung-Chieh Huang, Cheng-Hsu Chen, Mei-Chin Wen, Shang-Feng Tsai, Tung-Min Yu, Ya-Wen Chuang, Shih-Ting Huang, Shuo-Chun Weng, Mu-Chi Chung, Chia-Tien Hsu, Chun-Yi Wu, Chun-Te Huang, Tsai-Jung Wang, Hsien-Fu Chiu.

Investigation: Mei-Chin Wen.

**Supervision:** Ming-Ju Wu, Cheng-Hsu Chen.

**Formal analysis:** Shang-Feng Tsai, Tung-Min Yu, Ya-Wen Chuang, Shih-Ting Huang, Shuo-Chun Weng, Mu-Chi Chung, Chia-Tien Hsu, Chun-Yi Wu, Chun-Te Huang¸ Tsai-Jung Wang, Hsien-Fu Chiu.

## References

[1] United States Renal Data System. 2020 USRDS annual data report: epidemiology of kidney disease in the United States. Bethesda, MD: National Institutes of Health, National Institute of Diabetes and Digestive and Kidney Diseases. 2020.

[2] Hsu C-C Liao C-T Lin M-Y. Summary Report of the 2019 Annual Report on Kidney Disease in Taiwan. Acta Nephrologica. 2021;35:3–14.

[3] Department of Human Resources Development, National Development Council. Population projections for the Republic of China (Taiwan): 2020-2070. 2020. Available at: https://pop-proj.ndc.gov.tw/main_en/dataSearch.aspx?uid=78&pid=78&upn=8D038F3F06D3982D. [access date August 13, 2021].

[4] Lin YY Huang CS. Aging in Taiwan: building a society for active aging and aging in place. Gerontologist. 2016;56:176–83. 26589450 10.1093/geront/gnv107

[5] Nitta K Okada K Yanai M. Aging and chronic kidney disease. Kidney Blood Press Res. 2013;38:109–20. 24642796 10.1159/000355760

[6] Rivas Velasquez KM Hames E. Evaluation and management of the older adult with chronic kidney disease. Primary Care. 2014;41:857–74. 25439538 10.1016/j.pop.2014.08.006

[7] Okabayashi Y Tsuboi N Kanzaki G. Aging vs. hypertension: an autopsy study of sclerotic renal histopathological lesions in adults with normal renal function. Am J Hypertens. 2019;32:676–83. 31066457 10.1093/ajh/hpz040

[8] Hödlmoser S Winkelmayer WC Zee J. Sex differences in chronic kidney disease awareness among US adults, 1999 to 2018. PLoS One. 2020;15:e0243431e0243431. 33338051 10.1371/journal.pone.0243431 PMC7748269

[9] Hsiao K-C Lian J-D Wu S-W. Ten-year registry of native kidney biopsy from a single center in Taichung. Acta Nephrologica. 2012;26:68–73.

[10] Chiu HF Chen HC Lu KC Taiwan Society of Nephrology. Distribution of glomerular diseases in Taiwan: preliminary report of National Renal Biopsy Registry-publication on behalf of Taiwan Society of Nephrology. BMC Nephrol. 2018;19:6. 29320993 10.1186/s12882-017-0810-4 PMC5764016

[11] Bomback AS Herlitz LC Markowitz GS. Renal biopsy in the elderly and very elderly: useful or not? Adv Chronic Kidney Dis. 2012;19:61–7. 22449342 10.1053/j.ackd.2011.09.003

[12] Harmankaya O Okuturlar Y Kocoglu H. Renal biopsy in the elderly: a single-center experience. Int Urol Nephrol. 2015;47:1397–401. 26135198 10.1007/s11255-015-1035-8

[13] Chen Y-A Yang C-S. Renal biopsy in aged patients-12 years experience in Taiwan Cathay General Hospital. Acta Nephrologica. 2007;21:97–103.

[14] Molnár A Thomas MJ Fintha A. Kidney biopsy-based epidemiologic analysis shows growing biopsy rate among the elderly. Sci Rep. 2021;11:24479. 34966177 10.1038/s41598-021-04274-9 PMC8716536

[15] Perkowska-Ptasinska A Deborska-Materkowska D Bartczak A. Kidney disease in the elderly: biopsy based data from 14 renal centers in Poland. BMC Nephrol. 2016;17:194. 27884116 10.1186/s12882-016-0410-8 PMC5123353

[16] Shlipak MG Katz R Kestenbaum B. Rate of kidney function decline in older adults: a comparison using creatinine and cystatin C. Am J Nephrol. 2009;30:171–8. 19349699 10.1159/000212381 PMC2820322

[17] Helvaci O Korucu B Gonul I. Kidney biopsy in the elderly: diagnostic adequacy and yield. Int Urol Nephrol. 2021;53:105–9. 32940813 10.1007/s11255-020-02640-6

[18] Omokawa A Komatsuda A Nara M. Renal biopsy in patients aged 80 years and older: a single-center experience in Japan. Clin Nephrol. 2012;77:461–7. 22595388 10.5414/cn107368

[19] Marengoni A Angleman S Melis R. Aging with multimorbidity: a systematic review of the literature. Ageing Res Rev. 2011;10:430–9. 21402176 10.1016/j.arr.2011.03.003

[20] Denic A Glassock RJ Rule AD. Structural and functional changes with the aging kidney. Adv Chronic Kidney Dis. 2016;23:19–28. 26709059 10.1053/j.ackd.2015.08.004 PMC4693148

[21] Shiiki H Saito T Nishitani Y. Research Group on Progressive Renal Diseases in Japan. Prognosis and risk factors for idiopathic membranous nephropathy with nephrotic syndrome in Japan. Kidney Int. 2004;65:1400–7. 15086481 10.1111/j.1523-1755.2004.00518.x

[22] Choi JY Chin HJ Lee H. Idiopathic membranous nephropathy in older patients: clinical features and outcomes. PLoS One. 2020;15:e0240566e0240566. 33035278 10.1371/journal.pone.0240566 PMC7546503

[23] Cattran DC Kim ED Reich H. Membranous nephropathy: quantifying remission duration on outcome. J Am Soc Nephrol. 2017;28:995–1003. 27756808 10.1681/ASN.2015111262 PMC5328151

[24] Lee T Derebail VK Kshirsagar AV. Patients with primary membranous nephropathy are at high risk of cardiovascular events. Kidney Int. 2016;89:1111–8. 26924046 10.1016/j.kint.2015.12.041 PMC6787915

[25] Beck LH Jr. Bonegio RG Lambeau G. M-type phospholipase A2 receptor as target antigen in idiopathic membranous nephropathy. N Engl J Med. 2009;361:11–21. 19571279 10.1056/NEJMoa0810457 PMC2762083

